# A short and efficient synthesis of valsartan via a Negishi reaction

**DOI:** 10.3762/bjoc.6.27

**Published:** 2010-03-18

**Authors:** Samir Ghosh, A Sanjeev Kumar, G N Mehta

**Affiliations:** 1 Applied Chemistry Department, S.V. National Institute of Technology, Surat-395 007, India

**Keywords:** antihypertensive therapy, aryl bromide, Negishi coupling, tetrazole, valsartan

## Abstract

An efficient synthesis of the angiotensin-II inhibitor valsartan (Diovan^®^) is presented. Directed ortho-metalation of 5-phenyl-1-trityl-1*H*-tetrazole (**6**) and its Negishi coupling with aryl bromide **5** are the key steps of the synthesis. This method overcomes many of the drawbacks associated with previously reported syntheses.

## Introduction

**Figure 1:**
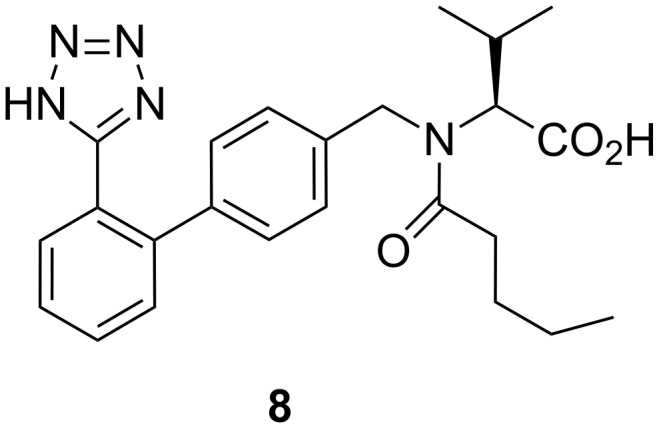
Valsartan.

Valsartan (Figure 1) is a member of a class of compounds known as angiotensin II (AT-II) receptor antagonists. This class combines effective anti-hypertensive activity with an excellent profile of safety and tolerability. Activation of AT-II receptors in the outer membrane of vascular smooth muscle cells of the heart and arteries causes the tissues to constrict. AT-II receptors are activated by the octapeptide AT-II. AT-II helps to maintain constant blood pressure [1] despite fluctuations in a person’s state of hydration, sodium intake and other physiological variables. AT-II also performs the regulatory tasks of inhibiting the excretion of sodium by the kidneys, inhibiting norephedrine re-uptake and stimulating aldosterone biosynthesis.

Valsartan [2] is therefore a non-peptide AT-II antagonist. By inhibiting the actions of AT-II on its receptors, valsartan prevents the increase of blood pressure produced by the hormone–receptor interactions. Hence, it is used in the treatment of cardiovascular complaints such as hypertension and heart failure. Comparative trial studies have shown that valsartan is as effective as angiotensin-converting enzyme (ACE) [3] inhibitors, calcium-channel blockers and α-blockers, and is generally better tolerated. Valsartan is marketed as the free acid under the trade name Diovan^®^. In addition, in combination with diuretics such as hydrochlorothiazide, valsartan offers specific advantages as an anti-hypertensive agent.

The formation of the aryl–aryl bond represents the key step in the synthesis of sartans: whilst the synthesis of losartan [4] as described in the literature makes use of Negishi [5–6] and Ullmann [7] couplings, the published methods for the preparation of valsartan utilize Suzuki–Miyaura couplings [8]. Of these, Negishi reactions have proved to be very efficient. However, the use of organozinc compounds provides better transmetalation activity than that obtained by the use of organoboron reagents as well as good chemoselectivity since most common functional groups are not attacked by organozinc species. Although preparations of several biphenyl ring systems related to valsartan have been reported, a number of challenges and some disadvantages - such as tedious reaction conditions, low yields and multistep sequences - still exist. Therefore, developing an efficient synthetic strategy with fewer steps that provides diverse access to these bioactive compounds is an important goal. In this paper, we report a new, concise and efficient synthesis of valsartan via Negishi coupling.

## Results and Discussion

**Scheme 1:**
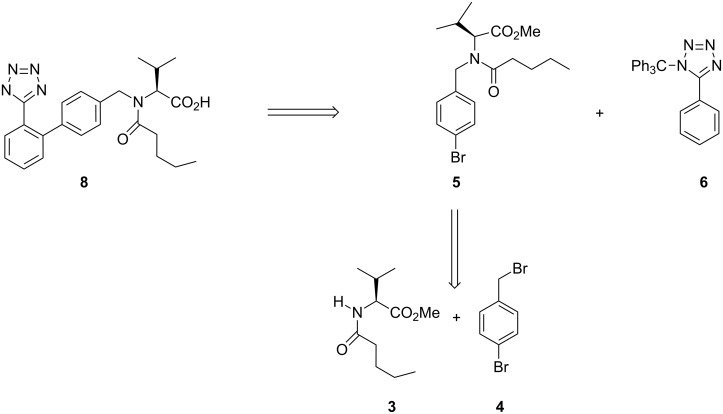
Retrosynthetic analysis of **8**.

From a retro-synthetic analysis (Scheme 1), compound **8** could be constructed via Negishi coupling from aryl bromide **5** and 5-phenyl-1-trityl-1*H*-tetrazole (**6**), which in turn could be obtained from commercially available benzonitrile. Aryl bromide **5** could be accessed by several discrete reactions of compound **3** and compound **4** via a nucleophilic substitution reaction.

**Scheme 2:**
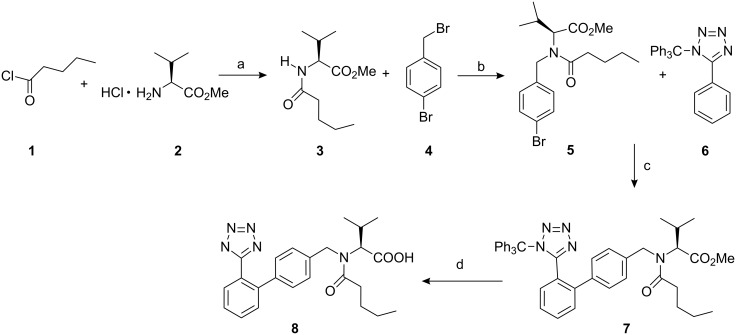
(a) Et_3_N, CH_2_Cl_2_, 0 °C, 95%; (b) NaH, THF, 70%; (c) *n*-BuLi, 25 °C, THF, anhyd ZnCl_2_, −20 °C, Q-phos, Pd(OAc)_2_, 75 °C, 2 h, 80%; (d) 3 N NaOH, MeOH, reflux, 90%.

As shown in Scheme 2, inexpensive and commercially readily available valeryl chloride **1** was coupled with L-valine methyl ester hydrochloride (**2)** in the presence of triethylamine in dichloromethane at 0 °C to afford methyl *N*-pentanoyl-L-valinate in 95% yield. Compound **3** was N-protected with 1-bromo-4-(bromomethyl)benzene in presence of sodium hydride in tetrahydrofuran to give methyl *N*-(4-bromobenzyl)-*N*-pentanoyl-L-valinate (**5**) [9] in 70% yield. Ortho-metalation of 5-phenyl-1-trityl-1*H*-tetrazole (**6**) [10] with *n*-butyllithium at 25 °C followed by treatment with zinc chloride at −20 °C gave the desired organozinc chloride compound. Coupling of the latter with aryl bromide **5** in presence of a catalytic amount of Q-phos and palladium acetate in tetrahydrofuran at 75 °C produced methyl *N*-pentanoyl-*N*-{[2'-(1-trityl-1*H*-tetrazol-5-yl)biphenyl-4-yl]methyl}-L-valinate (**7**) in 80% yield. Hydolysis of **7** with 3 N NaOH in methanol gave valsartan **8** [11].

## Conclusion

In summary, a highly efficient approach to the biphenyltetrazole structure of the AT-II antagonists has been developed which involves Negishi coupling of metalated 5-phenyl-1-trityl-1*H*-tetrazole. The method is commercially viable and applicable to plant scale production. This approach provides an industrial viable procedure for the synthesis of valsartan.

## Experimental

### Materials and instruments

All solvents and reagents were purchased from the suppliers and used without further purification. All non-aqueous reactions were performed in dry glassware under a dry nitrogen atmosphere. Organic solutions were concentrated under reduced pressure. Thin layer chromatography was performed on Merck precoated Silica-gel 60 F_254_ plates. ^1^H and ^13^C NMR spectra were recorded on a Varian Gemini 400 MHz FT NMR spectrometer using CDCl_3_ or DMSO-*d*_6_ as solvent. Chemical shifts are reported in δ ppm relative to TMS. Mass spectra were recorded on a Shimadzu LCMS-QP 800 LC-MS and AB-4000 Q-trap LC-MS/MS.

**Methyl *N*-pentanoyl-L-valinate (3).** Triethylamine (8.33 mL, 59.88 mmol) was added to a suspension of L-valine methyl ester hydrochloride **2** (5.0 g, 29.94 mmol) in dichloromethane (50 mL). Valeryl chloride **1** (3.95, 32.93 mmol) was then added at 0 °C and the mixture stirred at 25 °C for 1 h. Water (50 mL) was added to the reaction mixture and the organic layer separated and concentrated. The solid compound was triturated with heptanes (50 mL) to give an off white solid **3** (6.11 g, 95% yield). *R*_f_ = 0.6 (7:3; heptanes/EtOAc), ^1^H NMR (400 MHz, DMSO-*d*_6_): δ 8.01 (s, 1H), 4.12 (m, 1H), 3.59 (s, 3H), 2.48 (m, 2H), 2.13 (m, 2H), 1.95 (m, 1H), 1.45 (m, 3H), 1.25 (m, 5H), 0.86 (d, *J* = 4.4 Hz, 3H); ^13^C NMR (100 MHz, CDCl_3_): δ 173.2, 77.4, 56.8, 52.0, 36.2, 34.9, 31.2, 27.7, 22.2, 18.8; ESIMS: *m/z* calcd [M]^+^: 215; found: 216 [M+H]^+^.

**Methyl *N*-(4-bromobenzyl)-*N*-pentanoyl-L-valinate (5).** Sodium hydride dispersion (60%) in mineral oil (1.83 g, 46.51 mmol) was added to a solution of compound **3** (5.0 g, 23.25 mmol) and 1-bromo-4-(bromomethyl)benzene (**4**) (6.39 g, 25.58 mmol) in tetrahydrofuran (80 mL). The reaction mixture was refluxed for 1 h. After cooling, the mixture was diluted with ether (100 mL) and washed successively with saturated aq NH_4_Cl (50 mL) and water (100 mL). The organic layer was dried over Na_2_SO_4_ and concentrated in vacuum. The residue was chromatographed on silica gel. Elution with a mixture of heptanes and ethyl acetate (70:30) yielded the title compound **5** (6.25 g, 70%) as a colorless oil. *R*_f_ = 0.5 (7:3; heptanes/EtOAc), ^1^H NMR (400 MHz, DMSO-*d*_6_): δ 7.54 (d, *J* = 6.8 Hz, 2H), 7.29 (d, *J* = 8.8 Hz, 2H), 5.01 (s, 2H), 4.13 (m, 1H) 3.31 (s, 5H), 2.32 (t, *J* = 14.8 Hz, 2H) 1.50 (m, 2H), 1.93 (m, 1H), 1.24 (m, 3H), 1.22 (m, 3H), 0.83 (d, *J* = 7.6 Hz, 3H); ^13^C NMR (100 MHz, DMSO-*d*_6_): δ 173.9, 136.5, 133.1, 131.3, 121.1, 68.3, 52.5, 49.9, 34.2, 29.5, 27.2, 24.1, 23.2, 22.1, 19.2; ESIMS: *m/z* calcd [M]^+^: 384; found: 385 [M+H]^+^. HRMS (ESI): *m/z* calcd [M]^+^: 384.3079; found: 384.3085 [M]^+^.

**Methyl *N*-pentanoyl-*N*-{[2'-(1-trityl-1*H*-tetrazol-5-yl)biphenyl-4-yl]methyl}-L-valinate (7).** To a solution of 5-phenyl-1-trityl-1*H*-tetrazole (**6**) (2.0 g, 5.15 mmol) in THF (20 mL), *n*-BuLi (2.5 M in hexane) (2.5 mL, 6.18 mmol) was added at 25 °C under a N_2_ atmosphere. The reaction mixture was stirred at 25 °C for 1 h and then cooled to −20 °C. Anhydrous ZnCl_2_ (1.25 g, 9.27 mmol) was added to the reaction mixture and then stirred at −20 °C for 30 min. The reaction mixture was allowed to warm to 25 °C. Aryl bromide **5** (2.37 g, 6.18 mmol) followed by Q-phos (0.182 g, 0.25 mmol) and Pd(II)OAc (0.06 g, 0.25 mmol) was added to the reaction mixture. The reaction mixture was heated to reflux at 75 °C for 2 h. The reaction was monitored by TLC until the starting material was consumed. Water (30 mL) was added to the reaction mixture and extracted with ethyl acetate (3 × 50 mL). The ethyl acetate layer was separated and concentrated under vacuum. The residue was chromatographed on silica gel. Elution with a mixture of heptanes and ethyl acetate (70:30) gave the title compound **7** (2.84 g, 80%) as a white solid, mp 45–47 °C, *R*_f_ = 0.6 (7:3; heptanes/EtOAc), ^1^H NMR (400 MHz, DMSO-*d*_6_) δ 7.72 (d, J = 7.2 Hz, 1H), 7.60 (m, 1H), 7.51 (m, 1H), 7.42 (m, 1H), 7.36 (m, 11H), 6.98 (m, 1H), 6.88 (m, 7H), 4.62 (m, 2H), 3.25 (s, 3H), 3.17 (s, 1H), 2.23 (m, 2H), 2.01 (m, 1H), 1.34 (m, 2H), 1.19 (m, 3H), 1.01(m, 2H), 0.86 (d, 3H), 0.74 (d, 3H); ^13^C NMR (100 MHz, DMSO-*d*_6_) δ 173.8, 170.9, 170.6, 164.0, 141.6, 141.3, 139.1, 137.6, 130.9, 130.0, 129.3, 128.7, 128.3, 128.0, 127.9, 127.4, 126.2, 82.7, 62.3, 51.7, 48.6, 33.6, 32.6, 27.6, 22.5, 20.3, 19.7, 14.4; ESIMS: *m/z* calcd [M]^+^: 691; found: 692 [M+H]^+^;

***N*-Pentanoyl-*N*-{[2'-(1*H*-tetrazol-5-yl)biphenyl-4-yl]methyl}-L-valine (8).** To a solution of compound **7** (2 g, 2.89 mmol) in methanol (20 mL), 3 N NaOH (2.85 mL) was added and the mixture heated under reflux for 6 h. The progress of the reaction was monitored by TLC until the starting material was absent. The reaction mixture was concentrated under reduced pressure and the residue was diluted with EtOAc (100 mL) and distilled H_2_O (20 mL). Hydrochloric acid (2 N HCl) was added dropwise to the mixture until the pH reached 4.0. Then the organic phase was separated and the aqueous phase extracted with EtOAc (3 × 50 mL). The combined organic extracts were dried over anhydrous Na_2_SO_4_. Evaporation of the solvent gave the crude product (1.12 g, 90%). Recrystallization from EtOAc afforded the anticipated product valsartan **8**; mp 114–118 °C; ^1^H NMR (400 MHz, DMSO-*d*_6_): δ 12.6 (brs, 1H), 7.72 (m, 4H), 7.24 (m, 1H), 7.15 (m, 2H), 6.94 (m, 1H), 4.58 (m, 1H), 4.40 (m, 1H), 3.33 (m, 1H), 2.25 (m, 1H), 1.52 (m, 6H), 0.9 (m, 3H), 0.84 (m, 3H), 0.74 (m, 3H); ^13^C NMR (100 MHz, DMSO-*d*_6_): δ 174.0, 172.4, 171.8, 141.7, 138.2, 131.54, 131.1, 131.0, 129.3,128.8, 128.2, 127.4, 126.7, 70.3, 63.4, 49.9, 32.9, 28.05, 27.3, 22.2, 20.6, 14.2; ESIMS: *m/z* calcd [M]^+^: 435; found: 436 [M+H]^+^; HRMS (ESI): *m/z* calcd [M]^+^: 435.5187; found: 435.5125 [M]^+^
